# Supplementary material for: Lineage recording in monoclonal gastruloids reveals heritable modes of early development
Source: bioRxiv. 2025 Jul 16:2025.05.23.655664. Originally published 2025 May 23. Preprint. [Version 2] doi: 10.1101/2025.05.23.655664 (PMC12139830; doi:10.1101/2025.05.23.655664)
Supplement: Supplementary file 1 [file NIHPP2025.05.23.655664v2-supplement-2.pdf]

bioRxiv preprint doi: https://doi.org/10.1101/2025.05.23.655664; this version posted July 16, 2025. The copyright holder for this preprint (which was not certified by peer review) is the author/funder, who has granted bioRxiv a license to display the preprint in perpetuity. It is made available under aCC-BY 4.0 International license.

Supplementary Tables

Table S1. Marker genes used for mouse gastruloids cell type annotation.

bioRxiv preprint doi: https://doi.org/10.1101/2025.05.23.655664; this version posted July 16, 2025. The copyright holder for this preprint (which was not certified by peer review) is the author/funder, who has granted bioRxiv a license to display the preprint in perpetuity. It is made available under aCC-BY 4.0 International license.

Supplementary Note

Inferring tape integration events and patterns of edits

To achieve high resolution lineage trees, we developed an mESC line with a high DNA Typewriter Tape copy number via high MOI piggyBac integration. While such a system offers ample recording media necessary for diverse edit accumulation without exhaustion of recording capacity (*i.e.* saturation of editing sites on DNA Typewriter Tape), a major hurdle in the analysis of the DNA Typewriter data is that the Tape barcodes (TapeBCs) are not sufficient to define a unique genomic locus at which lineage information is recorded. This is due to the observation in our study that many or even most piggyBac integrated Tapes are duplicated in the genome, presumably due to excision/reintegration events before the transposase has diluted out. . The presence of duplicated Tapes presents a challenge for reconstructing lineages, as it becomes difficult to compare the patterns of edits observed for a given TapeBC across cells without knowing the underlying Tape locus from which they arose.

However, given that we have simultaneously recorded cell barcode information along with the expressed tape sequences, we can detect these duplication events as coexpressed tapes of differing patterns of edits. In other words, if a given cell has a sequential pattern of edits for a particular TapeBC of A-B-C but also expresses the same TapeBC with a pattern of D-E-F, this TapeBC is associated with at least two genomic integrations of DNA Typewriter Tape. Thus, we set out to devise a method that could infer the most likely structure of integration events per TapeBC into the genome as well as the patterns of edits that were generated from each integration locus. In this way, we can capture the true sequence of events that occur from the starting cell at each locus independently.

Determining consensus patterns of edits

We start from a table that is populated at each row by a unique sequence consisting of the CellBC, TapeBC, editing sites 1-6 (InsertBC(1)-(6)), and the number of UMIs that contain this exact sequence. Next, we filter this table for a given TapeBC in order to track its patterns of edits across all cells. We then compute a pairwise hamming distance matrix between each of the unique sequences at the edit sites. This distance matrix is then “clustered” by grouping all the unique sequences by exact matches and assigning them to a cluster if the cluster size would be greater than 100 UMIs. The remaining UMIs that had not been assigned to any cluster would be discarded as noise; this is analogous to a DBSCAN approach with a radius of 0. The consensus sequence for each cluster is refined into only the edited portions by searching for the presence of the edit key “GGAT” every 7 bases. This approach offers a few advantages: by pooling information across all cells, we are looking for patterns of edits that occur throughout the dataset. Additionally, the sequences that are not assigned to any cluster are likely generated from PCR or sequencing errors, and can be easily identified and thrown out.

Generating graph of edit pattern mutual exclusivity

Once the consensus patterns of edits are identified, we can then generate a cell-by-pattern matrix, with the values being the number of UMIs associated with each cell and pattern. This matrix is then row-normalized and log-transformed in order to more clearly accentuate differences between cells. Next, a binarization threshold is determined as the lowest point between the modes of the distribution of normalized values in the cell-by-pattern matrix. Binarizing this matrix allows us to generate a more interpretable matrix which represents whether or not a given pattern of edits is present in that cell for that TapeBC. This binarized matrix represents an intuitive way to think about multiple integrations; if a cell has a row sum of more than 1 (i.e. more than one pattern of edits), there are multiple integrations. However, the goal here is not to infer per-cell integrations, but

bioRxiv preprint doi: https://doi.org/10.1101/2025.05.23.655664; this version posted July 16, 2025. The copyright holder for this preprint (which was not certified by peer review) is the author/funder, who has granted bioRxiv a license to display the preprint in perpetuity. It is made available under aCC-BY 4.0 International license.

rather to infer the tape integration structure across all cells. For example, if pattern 1 and pattern 2 are both co-expressed in the same cells, they must have been generated from different loci.

To learn the structure of Tape integrations across all cells for a given TapeBC and assign patterns of edits to specific loci of integration, we performed the following analysis: We clustered this binary matrix using DBSCAN to group cells with the exact same groups of expressed patterns. Cells that were not assigned to any given cluster, usually due to dropout, were assigned to a cluster using a KNN classifier in normalized log-UMI space. Each cluster thus represents a group of cells for which a given pattern is expressed. For each cluster, given its representative binary vector of whether each pattern of edits is expressed, we can then generate a matrix of mutual exclusivity by computing the pairwise cosine distance between this vector and itself. This generates an adjacency matrix which in turn implies a graph whose nodes represent patterns of edits and whose edges represent mutual exclusivity between that pair of patterns in the same cells. The cosine metric was chosen because it only draws an edge between patterns if a 1 is present for both patterns, rather than a 0 in both. We compute such graphs for each cluster of cells and then intersect them all to generate a single representative graph across all cells. The assumption here is that if two patterns are co-expressed in at least one cluster, they must be mutually exclusive with respect to tape locus in all cells. That is to say, those patterns of edits must have been generated at separate loci by definition.

Grouping patterns into loci

Given this graph, how can we determine how to group patterns into shared loci? If such a graph were complete, that is to say fully connected, it would imply that each pattern of edits is never co-expressed and therefore there is only one locus from which all patterns were generated. We can extend this idea to our observed graphs: a complete subgraph (or clique) within the graph represents a putative locus (or Tape integration event). Therefore, the problem can be formulated in terms of finding a node partition of the graph such that each induced subgraph is a clique. However, we must constrain this problem with several biological considerations. The most likely explanation is one that minimizes the number of inferred integration events; thus, we pick the solution that minimizes the number of cliques. Given that there may be multiple equivalent solutions under this metric, we introduce edge weights as the number of shared consecutive edits between patterns. In this way, cliques with higher sums of edge weights have more related patterns and are therefore more likely to represent patterns that were generated from the same locus. To summarize, the problem becomes finding a clique partition of the graph that: 1) minimizes the number of cliques, and 2) maximizes the sum of edge weights across all cliques.

The problem of finding minimal clique partitions cannot be realistically achieved through exhaustive search on large datasets. However, the problem can be flipped on its head. The complement of the graph, generated by inverting all the edges, can be “colored” with a minimal number of colors using greedy graph coloring approaches. Graph coloring refers to a problem where nodes are assigned colors such that no two neighboring nodes can share the same color. Thus, the color assignments of the graph complement correspond to clique memberships in the original graph. However, since we need to also optimize for the sum of edge weights in the original graph, we run the graph coloring algorithm a large number of times with randomized node orderings and select the solution which produces the fewest number of cliques and the highest sum of edge weights retained.

Each of these inferred cliques then represents a locus of integration for that specific TapeBC, as well as grouping the exact patterns that belong at that locus. From this, we can then generate a final matrix with cells as the rows and inferred loci as the columns. The detected patterns per cell are then stored in the columns according to the clique groupings. This final matrix is then used as the basis for calculating pairwise lineage distances between cells and ultimately for generating phylogenies with UPGMA.

bioRxiv preprint doi: https://doi.org/10.1101/2025.05.23.655664; this version posted July 16, 2025. The copyright holder for this preprint (which was not certified by peer review) is the author/funder, who has granted bioRxiv a license to display the preprint in perpetuity. It is made available under aCC-BY 4.0 International license.

SUPPLEMENTARY FIGURES

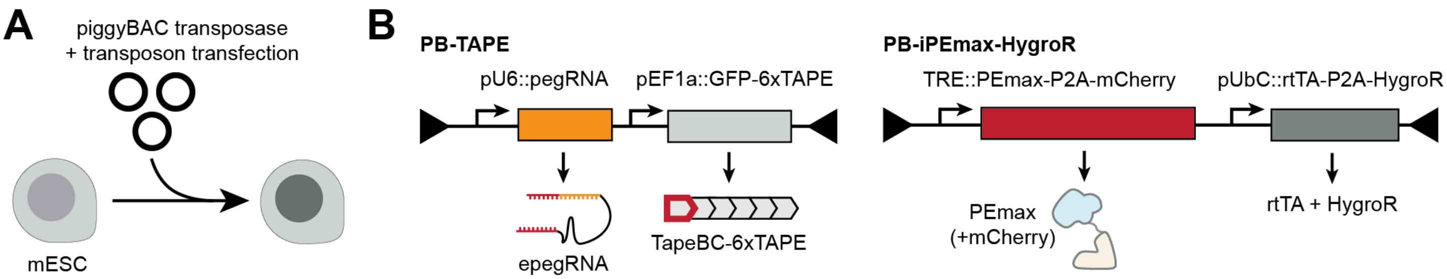

Figure S1. Generation of mESCs with DNA Typewriter Tape.
(A) Schematic of transfecting mESC with three different kinds of DNA plasmids: transposon bearing DNA Typewriter Tape (PB-TAPE), transposon bearing Dox-inducible Prime Editor expression cassette (PB-iPEmax-HygroR), and plasmid expressing piggyBac transposase. **(B)** Architecture of two transposons: PB-TAPE contains a human U6 promoter driving epegRNA expression (pU6::epegRNA, where epegRNA inserts one of 64 possible NNNGGA sequences to DNA Typewriter Tape targets) and human EF1a promoter driving GFP-6xTAPE expression (pEF1a::GFP-6xTAPE), where 12-bp (NNNNNAANNNNN) TapeBC and 6xTAPE (DNA Typewriter Tape with 6 sequential editing sites) embedded in 3'-UTR of eGFP-encoding transcript. PB-iPEmax-HygroR contains a Dox-inducible promoter driving expression of PEmax-P2A-mCherry (TRE::PEmax-P2A-mCherry) and a human ubiquitin C promoter driving rtTA-P2A-HygroR expression. Dox-induction of modified cells result in expression of PEmax that binds to epegRNA and targets 6xTAPE for random sequential editing to generate a heritable DNA barcode used for lineage tracing and lineage tree reconstruction.

bioRxiv preprint doi: https://doi.org/10.1101/2025.05.23.655664; this version posted July 16, 2025. The copyright holder for this preprint (which was not certified by peer review) is the author/funder, who has granted bioRxiv a license to display the preprint in perpetuity. It is made available under aCC-BY 4.0 International license.

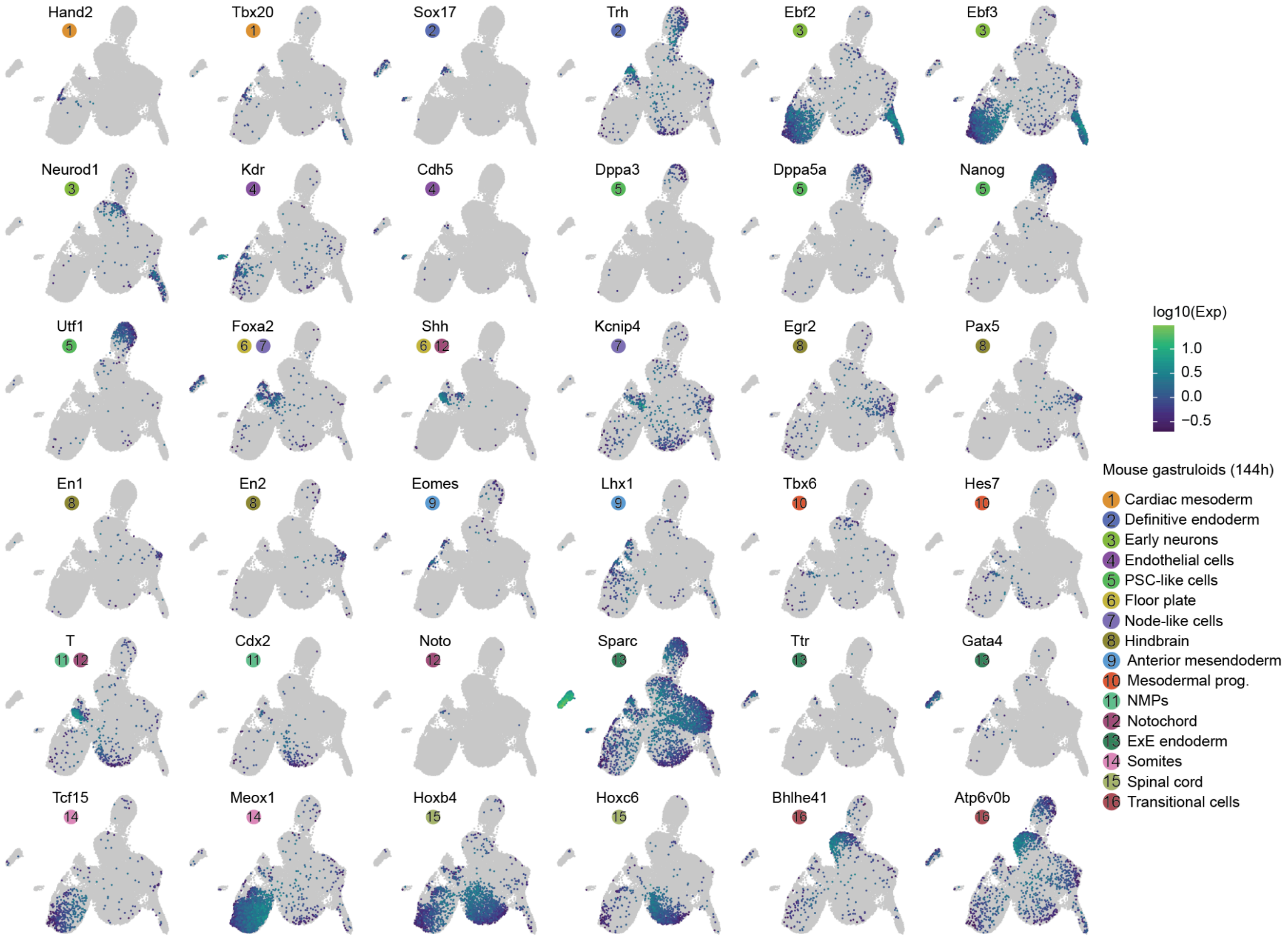

Figure S2. Transcriptional heterogeneity in mouse gastruloids.

The same UMAP as in **Figure 2B**, colored by gene expression of marker genes for each cell cluster. References for marker genes are provided in **Table S1**.

bioRxiv preprint doi: https://doi.org/10.1101/2025.05.23.655664; this version posted July 16, 2025. The copyright holder for this preprint (which was not certified by peer review) is the author/funder, who has granted bioRxiv a license to display the preprint in perpetuity. It is made available under aCC-BY 4.0 International license.

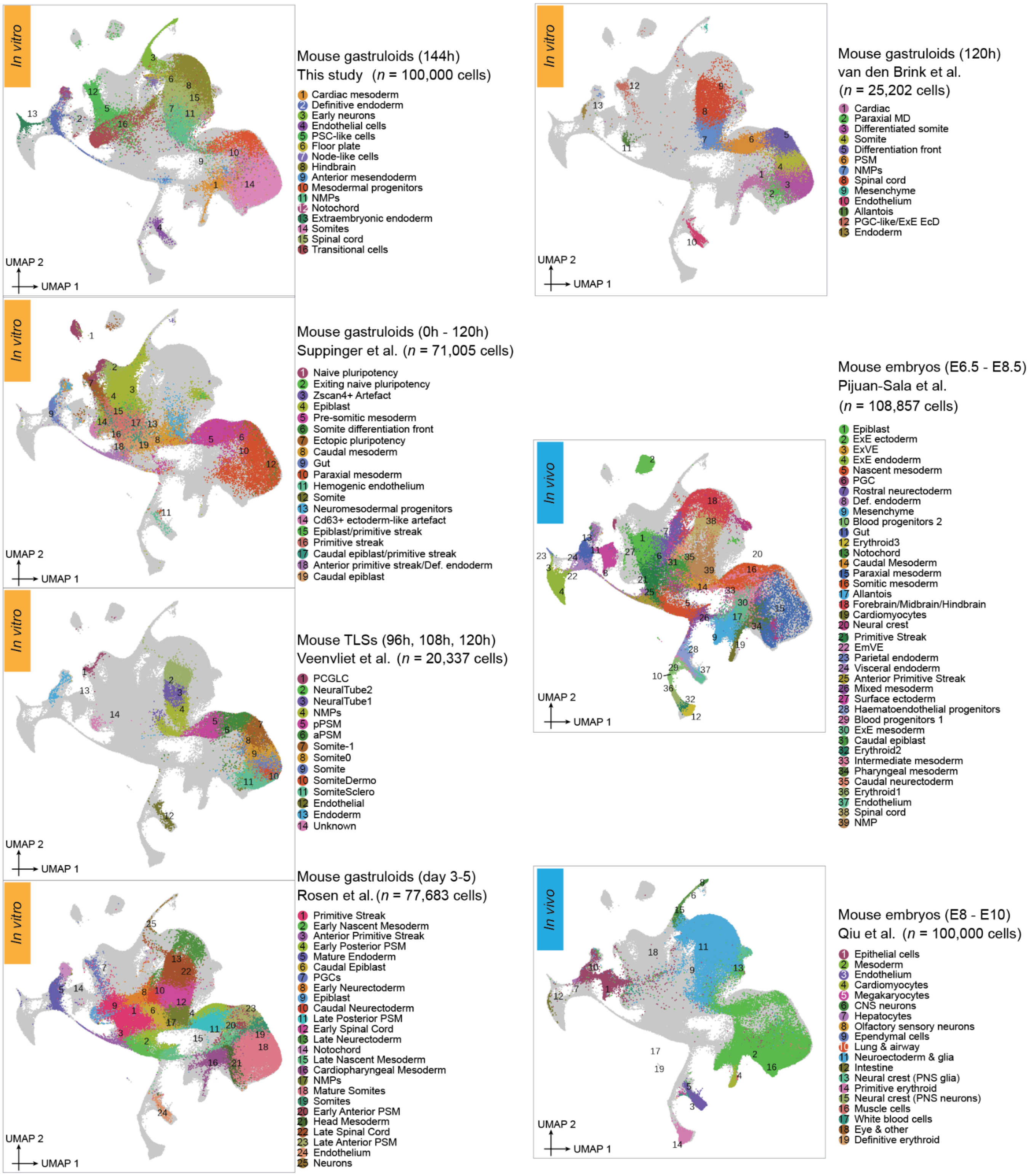

Figure S3. Integrating & co-embedding cells from seven studies of mouse gastruloids or embryos. UMAP visualization of 503,084 co-embedded cells from seven datasets, including mouse gastruloids from this study, four published mouse gastruloid studies[25,29,32,33], and two studies of mouse embryos during gastrulation (E6.5-E8.5)[31] or early somitogenesis (E8-E10)[30], after batch correction[53]. The same UMAP is shown seven times, with colors highlighting cells from each dataset. To align with the cell counts in the other datasets, sci-RNA-seq3 profiles from this study (top left) and mouse embryos early somitogenesis[30] (bottom right) were randomly subsampled to 100,000 cells each.

bioRxiv preprint doi: https://doi.org/10.1101/2025.05.23.655664; this version posted July 16, 2025. The copyright holder for this preprint (which was not certified by peer review) is the author/funder, who has granted bioRxiv a license to display the preprint in perpetuity. It is made available under aCC-BY 4.0 International license.

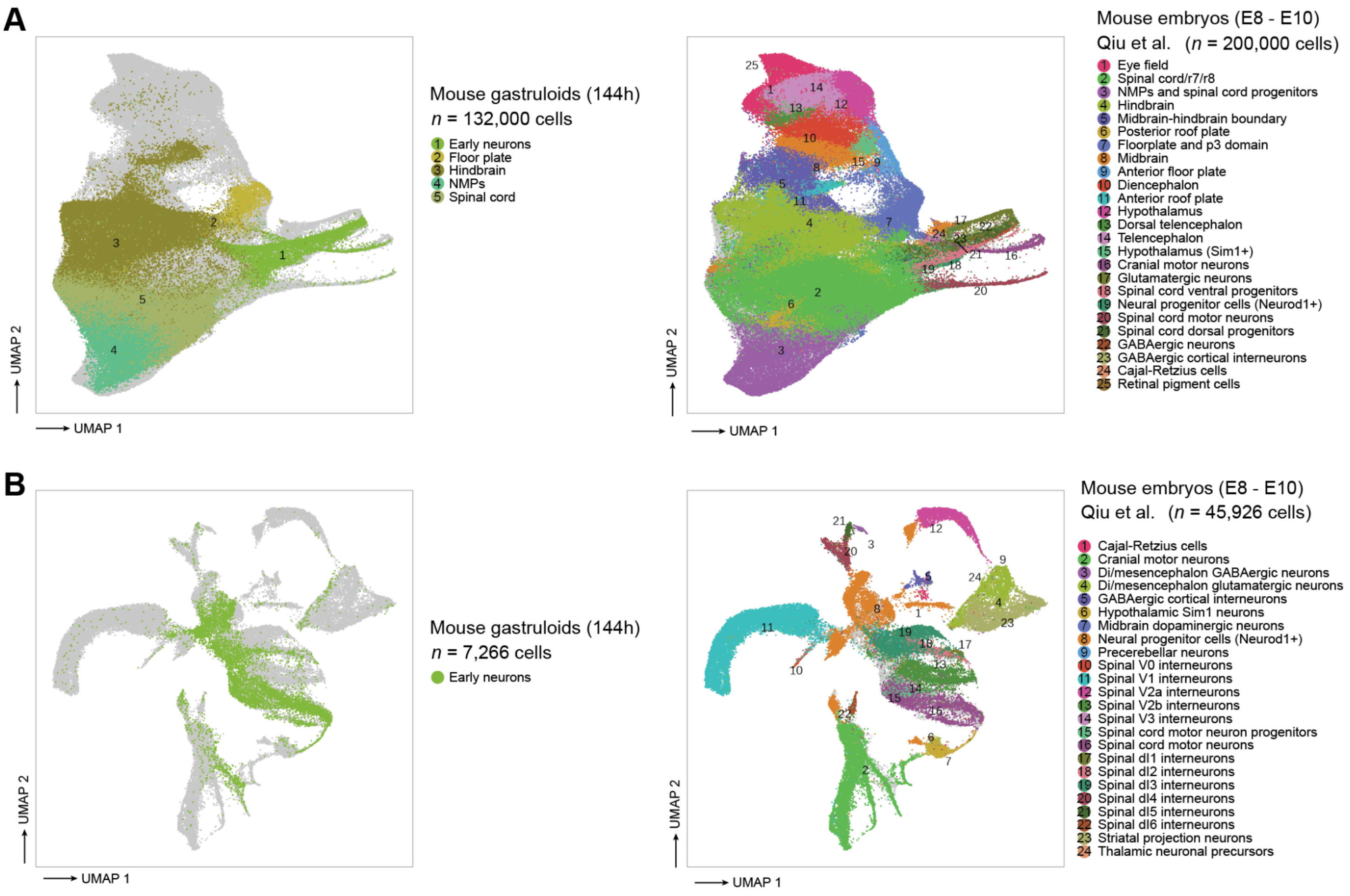

Figure S4. Integrating & co-embedding patterned neuroectodermal and neuronal cells from mouse gastruloids and embryos.

(A) UMAP visualization of single cell transcriptional profiles of 332,000 nuclei annotated as patterned neuroectodermal cell types from either 144 hr mouse gastruloids (left; this study) or mouse embryos during early somitogenesis (E8-E10)[30] (right), after batch correction[53]. The same UMAP is shown twice, with colors highlighting cells from each dataset. Cells from the mouse embryo study were randomly subsampled to 200,000. **(B)** Same as panel **A**, but for 53,192 nuclei annotated as neuronal cell types in the same two studies.

bioRxiv preprint doi: https://doi.org/10.1101/2025.05.23.655664; this version posted July 16, 2025. The copyright holder for this preprint (which was not certified by peer review) is the author/funder, who has granted bioRxiv a license to display the preprint in perpetuity. It is made available under aCC-BY 4.0 International license.

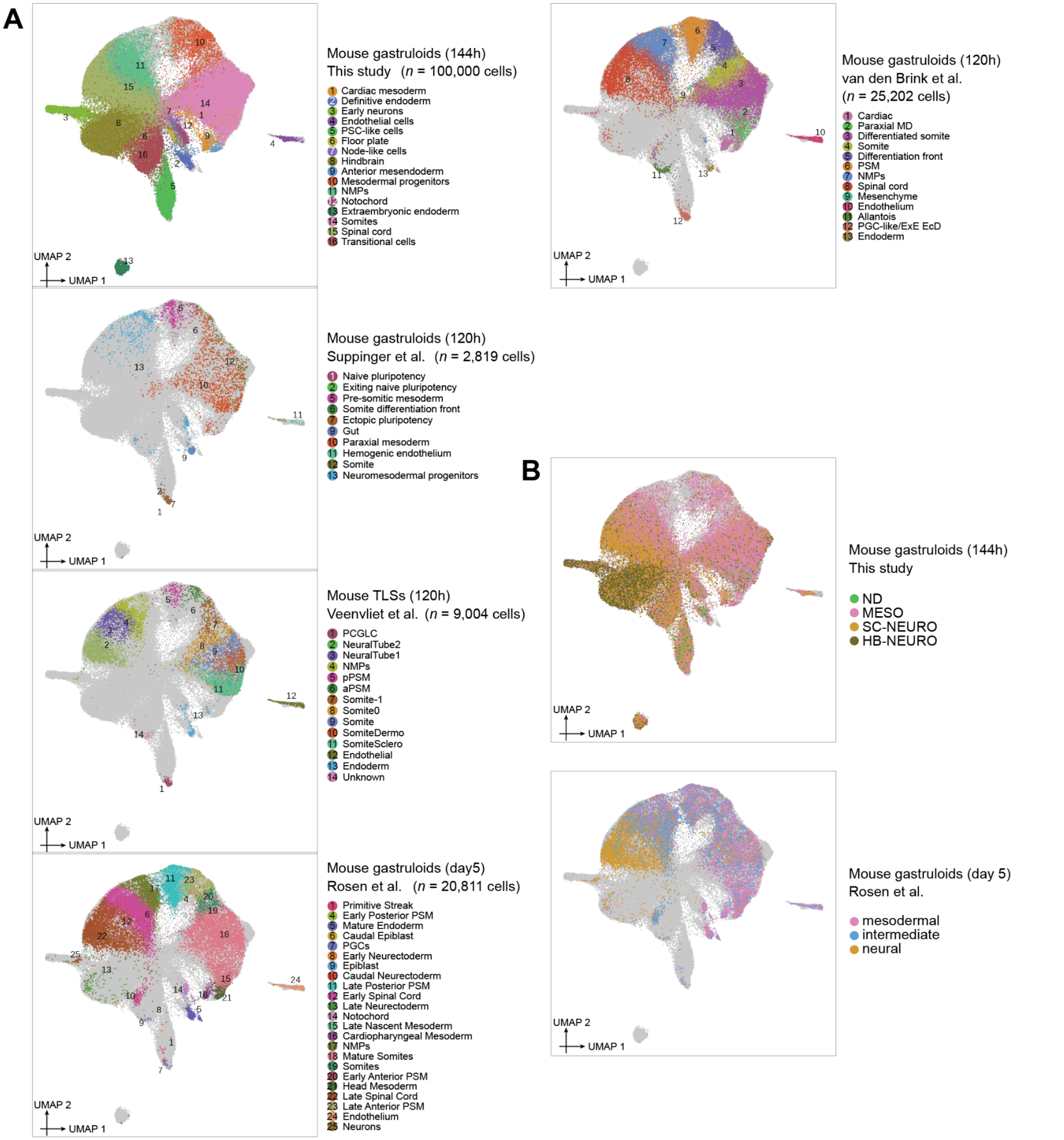

Figure S5. Integrating & co-embedding cells from mouse gastruloids from this study (144 hrs) and four other studies (120 hr time point only).

(A) UMAP visualization of 157,836 co-embedded cells from five datasets, including mouse gastruloids from this study (144 hours), and four other published mouse gastruloid studies (120 hours)[25,29,32,33], after batch correction[53]. The same UMAP is shown five times, with colors highlighting cells from each dataset. To align with the cell counts in the other datasets, cells from the current study (top left) were randomly subsampled to 100,000 cells each. **(B)** The same UMAP as shown in panel **A** is displayed twice, with colors highlighting cells from the four gastruloid groups delineated in this study (top), or from the clusters delineated by Rosen et al. (2022)[32].

bioRxiv preprint doi: https://doi.org/10.1101/2025.05.23.655664; this version posted July 16, 2025. The copyright holder for this preprint (which was not certified by peer review) is the author/funder, who has granted bioRxiv a license to display the preprint in perpetuity. It is made available under aCC-BY 4.0 International license.

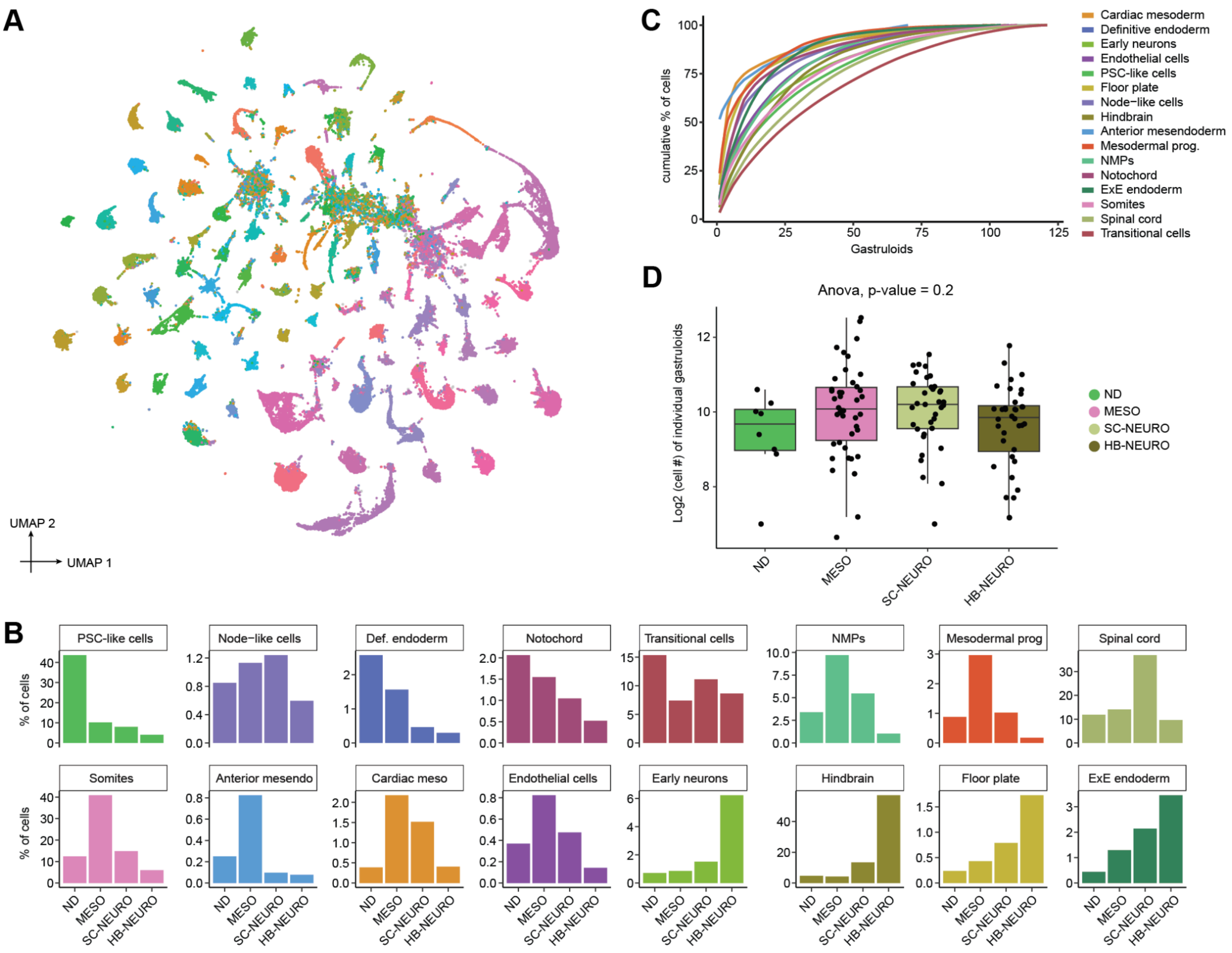

Figure S6. Assigning cells & assessing heterogeneity across 121 monoclonal mouse gastruloids.
(A) 2D UMAP visualization of 154,988 cells (with ≥3 TapeBCs detected) derived from 121 monoclonal mouse gastruloids. Dimensionality reduction was performed on a TapeBC × cell matrix with read counts as values, using PCA followed by UMAP purely for visualization purposes. Cells are colored according to the gastruloids to which they were assigned. Unassigned cells (4%) are depicted in gray. **(B)** The proportions of sixteen cell types for individual gastruloids were calculated, and the average values within each of the four groups (ND, MESO, SC-NEURO, HB-NEURO) are presented. **(C)** For each cell type, the cumulative percentage of cells was plotted across 121 gastruloids, ranked by the abundance of that cell type from highest to lowest. **(D)** Log2 number of cells assigned to individual gastruloids (n = 121) for each of the four groups. Boxplots represent IQR (25th, 50th, 75th percentile) with whiskers representing 1.5× IQR. ND: non-differentiating, MESO: somite-like, SC-NEURO: spinal cord-like, or HB-NEURO: hindbrain-like.

bioRxiv preprint doi: https://doi.org/10.1101/2025.05.23.655664; this version posted July 16, 2025. The copyright holder for this preprint (which was not certified by peer review) is the author/funder, who has granted bioRxiv a license to display the preprint in perpetuity. It is made available under aCC-BY 4.0 International license.

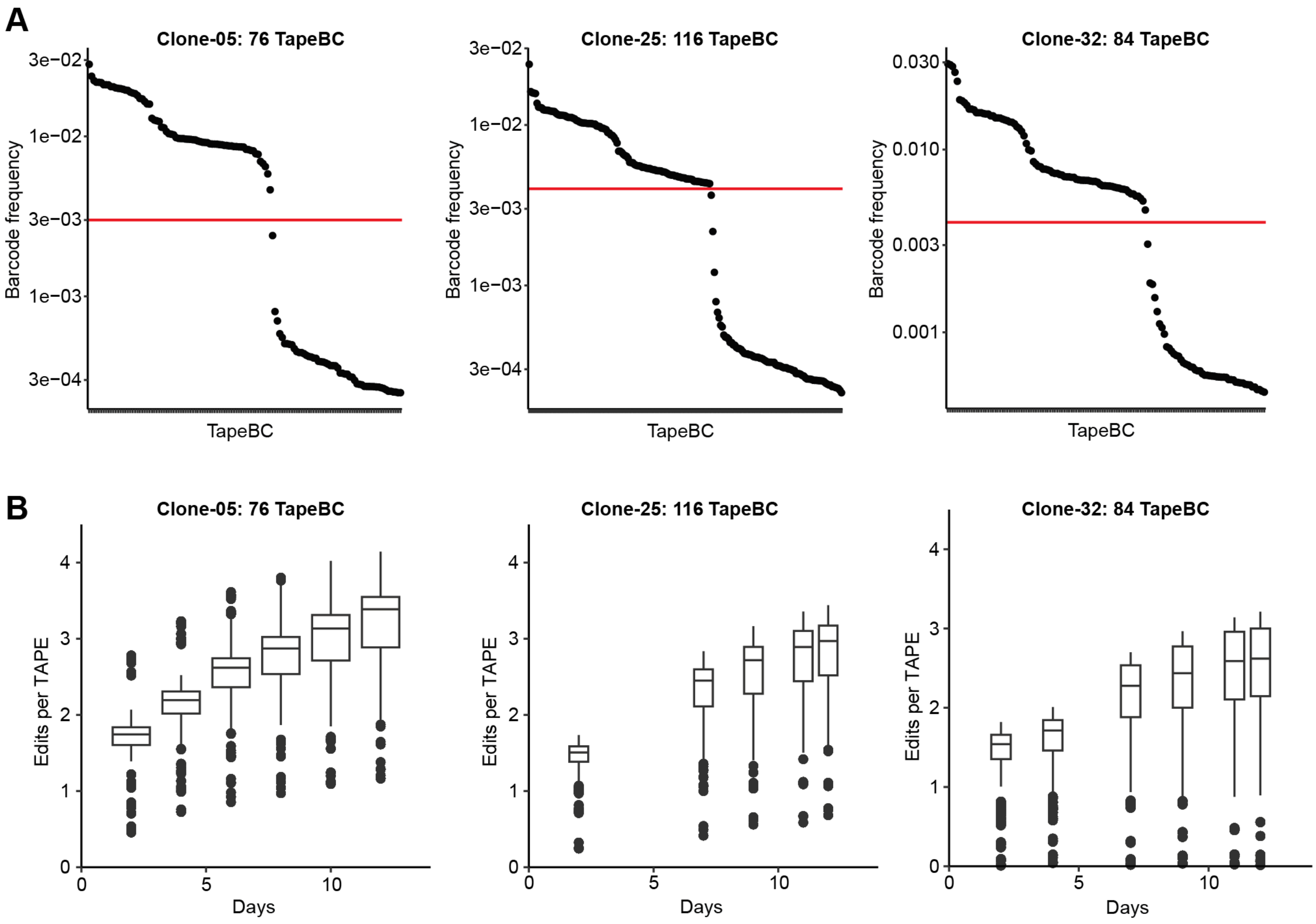

Figure S7. Characterization of three monoclonal mESC cell lines for recording capacity for DNA Typewriter-based lineage recording.

(A) TapeBC barcodes from each of three monoclonal mESC cell lines were PCR amplified and sequenced from genomic DNA. Frequency thresholds (red line) to define bona fide TapeBC barcodes were set as shown, resulting in estimates of 76, 116, and 84 DTT/epegRNA integrations for Clone-05, Clone-25, and Clone-32, respectively. Note that these are lower bounds, as some integrants are duplicated by piggyBac excision and re-integration events[28,34]. **(B)** The accumulation of editing at DTT in each monoclonal mESC cell line with continuous doxycycline (Dox) treatment to induce PEmax expression. 100 ng/mL Dox was added to cells at Day 0, and cell lines were collected at multiple time points of 2-3 days in interval, depending on the availability of cells at the time of the passaging and collection. The experiment was terminated at Day 12, which is equivalent to the last time point in the monoclonal gastruloid induction protocol. Boxplot represents IQR (25th, 50th, 75th percentile) with whiskers representing 1.5× IQR.

bioRxiv preprint doi: https://doi.org/10.1101/2025.05.23.655664; this version posted July 16, 2025. The copyright holder for this preprint (which was not certified by peer review) is the author/funder, who has granted bioRxiv a license to display the preprint in perpetuity. It is made available under aCC-BY 4.0 International license.

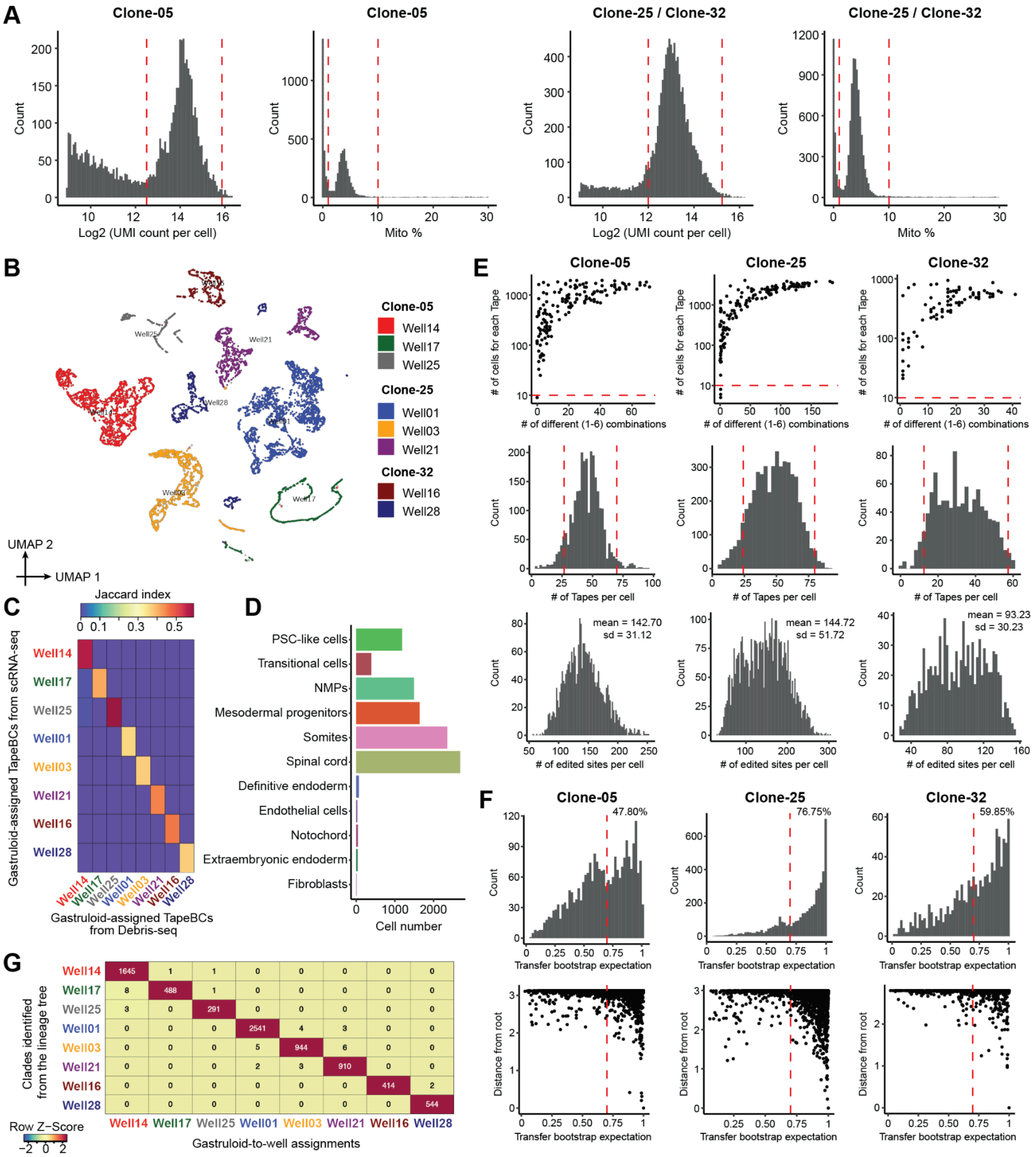

Figure S8. Quality control for scRNA-seq and cell-to-gastruloid assignments.

(A) Histograms of log2(UMI count) per cell and the proportion of reads mapping to mitochondrial chromosomes (Mito%) per cell, derived from scRNA-seq data (10X Genomics) from Lane 1 (left, Clone-05) and Lane 2 (right, Clones-25 & 32). Cells with log2(UMI counts) <12.5 in Lane 1 or <12 in Lane 2 were excluded, as were those in the top 0.5% of total UMI

bioRxiv preprint doi: https://doi.org/10.1101/2025.05.23.655664; this version posted July 16, 2025. The copyright holder for this preprint (which was not certified by peer review) is the author/funder, who has granted bioRxiv a license to display the preprint in perpetuity. It is made available under aCC-BY 4.0 International license.

counts (red vertical lines) and those with Mito% >10% or <1% (red vertical lines). **(B)** 2D UMAP visualization of 8,547 cells, with ≥2 TapeBCs detected and TapeBC UMIs ≥10. Dimensionality reduction was performed with PCA on a TapeBC × cell matrix with read counts as values, followed by UMAP solely for visualization. Cells are colored by their assignment to one of eight monoclonal gastruloids. Unassigned cells (2%) are depicted in light gray. **(C)** Jaccard similarities between the TapeBCs detected in cells assigned to each gastruloid (row; detected in ≥5% of the cells within that gastruloid) vs. TapeBCs detected by Debris-seq of each well (column). Only TapeBCs detected in both scRNA-seq and Debris-seq were included in these calculations. **(D)** Frequency of each cell type across 9,929 cells. **(E)** Filtering based on DTTs detected from Clone-05 (left), Clone-25 (middle), and Clone-32 (right): Top: The number of cells detected for each DTT (y-axis) is plotted against the number of different InsertBC(1-6) combinations (x-axis). DTTs with a cell count >10 (red horizontal lines) were retained. Middle: Histograms of the numbers of post-filtering DTTs detected per cell. Cells outside the range of mean - 1.5 × SD to mean + 2 × SD were excluded (red vertical lines). Bottom: Post-filtering histograms of the total number of edited sites per cell, with the mean and standard deviation indicated. **(F)** We performed a bootstrapping analysis to evaluate the robustness of the trees shown in **Figure 3D**, composed of 2,438 tips & 2,437 clades in Clone-05, 4,418 tips & 4,417 clades in Clone-25, and 960 tips & 959 clades in Clone-32. For each of 100 bootstraps, an equal number of DTTs (each containing six edit sites) was resampled with replacement to reconstruct a new tree. Clade concordance was determined by comparing each bootstrap tree to the original and calculating transfer bootstrap expectation (TBE)[35]. Briefly, the TBE of each clade in the original tree was calculated by determining the minimal transfer distance across all clades in the bootstrap tree, followed by computing the mean values over the 100 bootstraps. Here we show a histogram of the TBEs of clades (top), and a scatter plot of TBE against the distance from the root for each clade (bottom). Dashed red vertical lines indicate a TBE cutoff of 0.7, with values greater than that corresponding to moderate to strong support[35]. **(G)** The numbers of cells in each of ten major clades (with two clades combined for Well17 and Well01, respectively) of the cell lineage tree shown in **Figure 3D** plotted against the well assignments of those same cells.

bioRxiv preprint doi: https://doi.org/10.1101/2025.05.23.655664; this version posted July 16, 2025. The copyright holder for this preprint (which was not certified by peer review) is the author/funder, who has granted bioRxiv a license to display the preprint in perpetuity. It is made available under aCC-BY 4.0 International license.

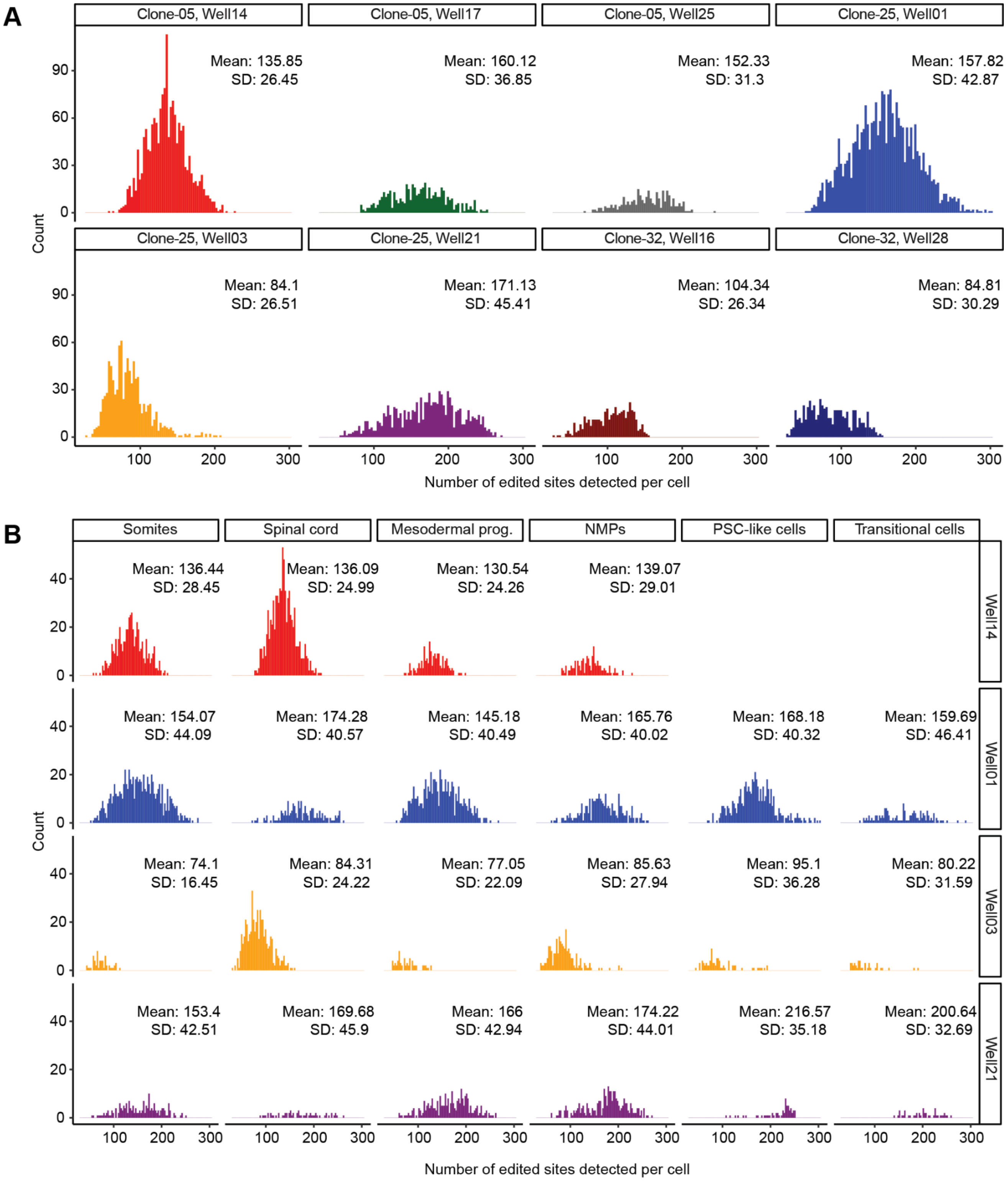

Figure S9. Numbers of edited sites in DTT detected per cell, across gastruloids and cell types.
(A) To systematically compare the overall activity of PEmax between gastruloids and cell lines, for each DTT observed in each of the 7,816 cells used to reconstruct the lineage tree, we counted the total number of edited sites observed in each cell. Histograms of these counts are shown, together with the mean and standard deviation, for each of the eight gastruloids. **(B)** Same as panel **A**, but for the four gastruloids with the most sampled cells, and further splitting these out by the six most abundant cell types.

bioRxiv preprint doi: https://doi.org/10.1101/2025.05.23.655664; this version posted July 16, 2025. The copyright holder for this preprint (which was not certified by peer review) is the author/funder, who has granted bioRxiv a license to display the preprint in perpetuity. It is made available under aCC-BY 4.0 International license.

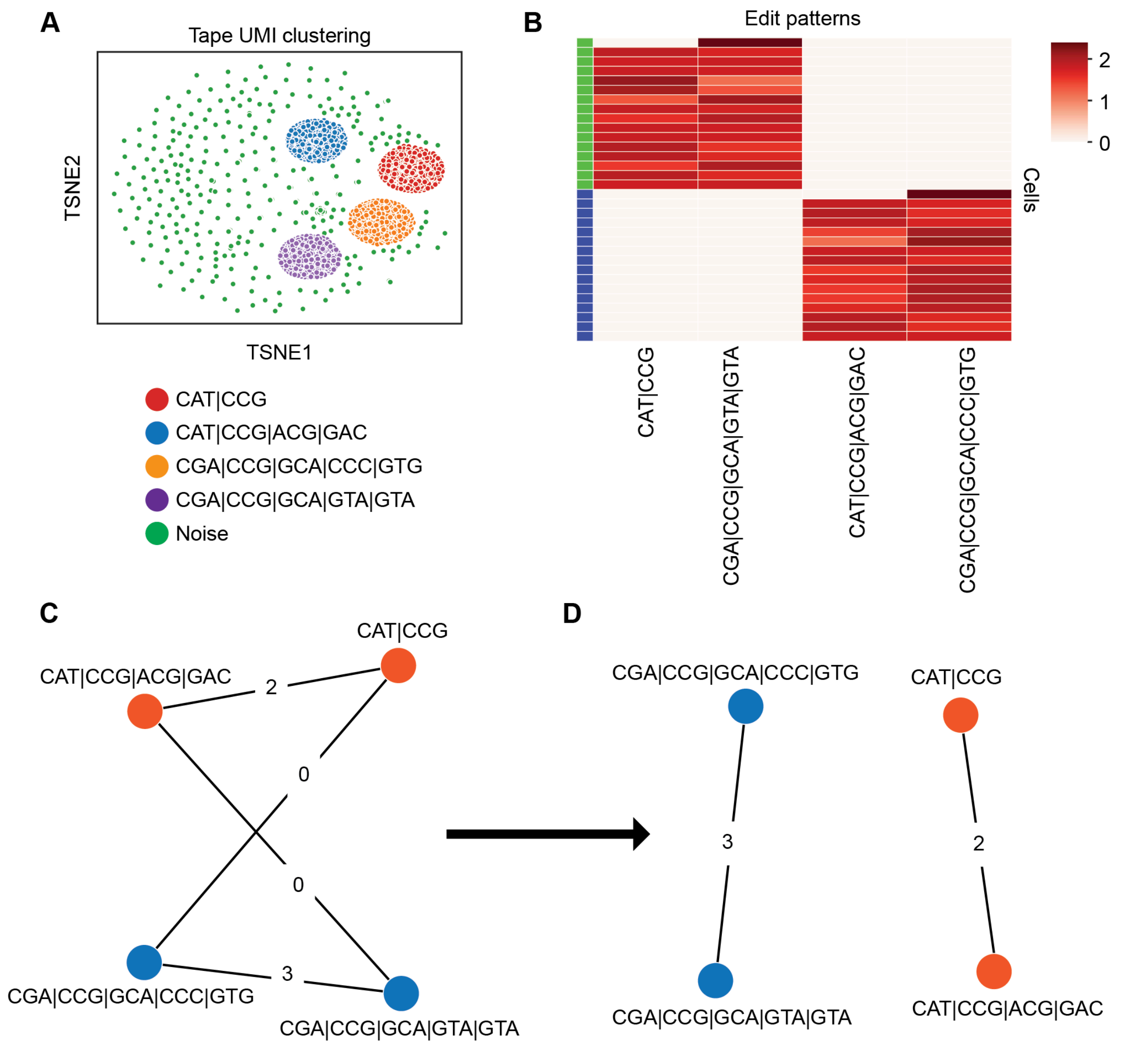

Figure S10. Disentangling TapeBCs that are integrated at multiple genomic locations.
We relied on piggyBac transposition at a high MOI in order to obtain a large number of DTTs in each monoclonal cell line. Each DTT is associated with a degenerate TapeBC that facilitates the assignment of DTT-derived sequencing reads to specific integrations (**Figure S1B**). However, a major challenge is that due to piggyBac excision and re-integration events[28,34] during the establishment of the monoclonal cell line, a given TapeBC might be associated with two or more independent integrations, confounding lineage reconstruction. Here we present an example vignette of our strategy for disentangling such duplicated TapeBCs by relying on the patterns in which their associated edits either do (consistent with deriving from separate integrations) or don't (consistent with deriving from the same integration) appear in the same cells. Further details on our algorithmic strategy are presented in a **Supplementary Note**. This example vignette is for a specific TapeBC from a subset of 32 cells with one major bifurcation into two clades. **(A)** t-SNE depicting clusters of DTT UMIs across all 32 cells. The labels for each cluster represent a unique pattern of edits. Each 3N edit is separated by a "|". UMIs not assigned to any cluster were discarded as likely noise from PCR or sequencing errors. **(B)** After identifying consensus edit patterns, we generated a cell-by-pattern matrix with UMI counts, row-normalized and log-transformed to highlight differences between cells. This matrix was then binarized (not shown) and the rows were clustered into two groups of cells. **(C)** The representative mutual exclusivity graph for this dataset of patterns is shown. An edge is drawn between two patterns of edits if and only if those two patterns of edits are never co-expressed in any cluster of cells in the previous matrix. Edge weights indicate the number of shared sequential edits between mutually exclusive patterns. **(D)** Graph coloring on the complement of the mutual exclusivity graph reveals the two most likely groups of mutually exclusive patterns. These groupings correspond to unique DTT integration loci of the same TapeBC.

bioRxiv preprint doi: https://doi.org/10.1101/2025.05.23.655664; this version posted July 16, 2025. The copyright holder for this preprint (which was not certified by peer review) is the author/funder, who has granted bioRxiv a license to display the preprint in perpetuity. It is made available under aCC-BY 4.0 International license.

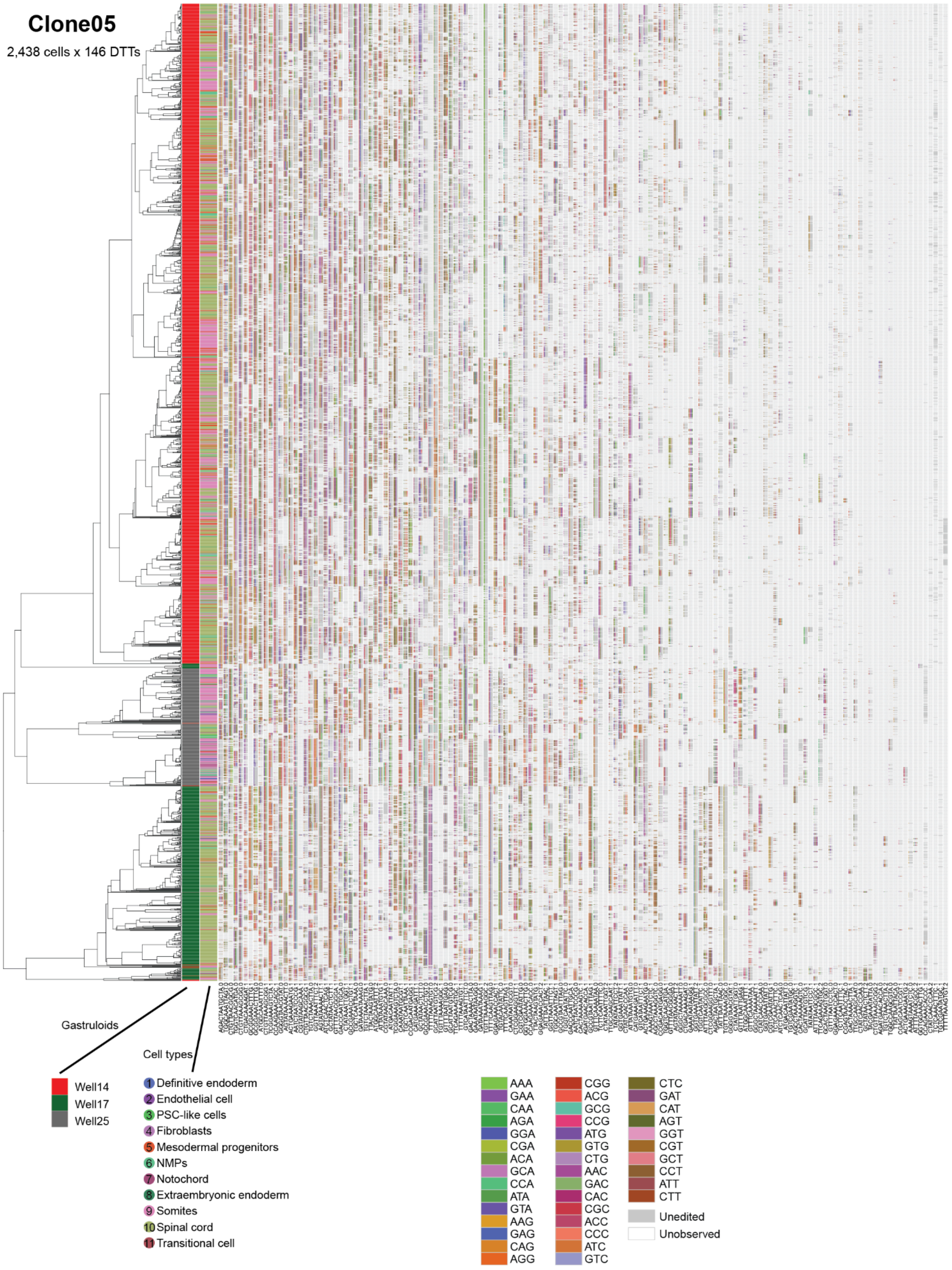

Figure S11. A cell-by-DTT matrix of Clone-05.

Left: The left dendrogram shows the phylogenetic tree of 2,438 cells from Clone-05 based on cell-cell distances, the colors of the middle bar correspond to 3 gastruloids to which each cell was assigned, and the colors of the right bar correspond to the 11 annotated cell types. **Right:** Edits at each site across 146 DTTs, ordered by total number of edited sites from high (left) to low (right). Observed, unedited sites are colored grey, while unobserved DTTs are colored white.

bioRxiv preprint doi: https://doi.org/10.1101/2025.05.23.655664; this version posted July 16, 2025. The copyright holder for this preprint (which was not certified by peer review) is the author/funder, who has granted bioRxiv a license to display the preprint in perpetuity. It is made available under aCC-BY 4.0 International license.

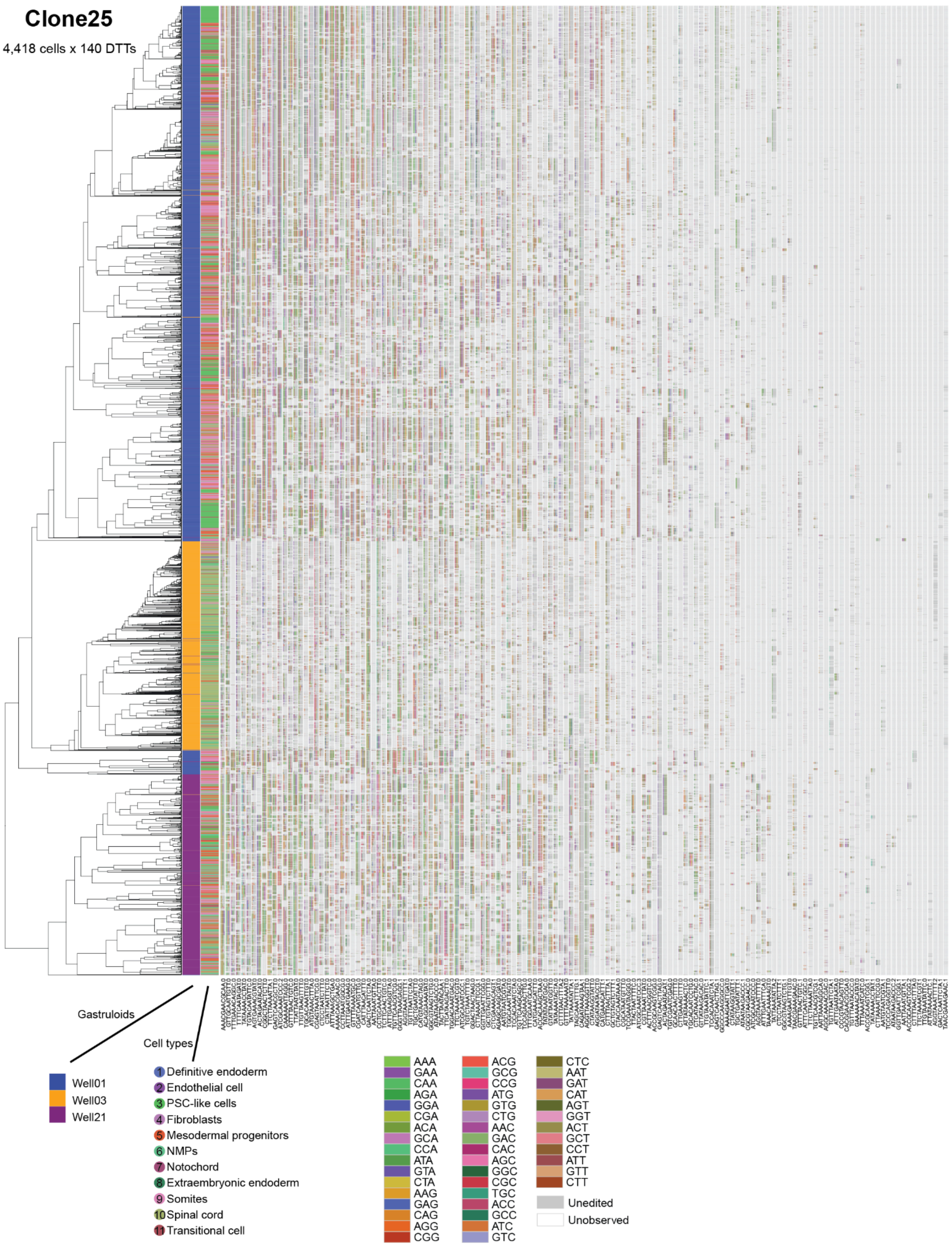

Figure S12. A cell-by-DTT matrix of Clone-25.

Left: The left dendrogram shows the phylogenetic tree of 4,418 cells from Clone-25 based on cell-cell distances, the colors of the middle bar correspond to 3 gastruloids to which each cell was assigned, and the colors of the right bar correspond to the 11 annotated cell types. **Right:** Edits at each site across 140 DTTs, ordered by total number of edited sites from high (left) to low (right). Observed, unedited sites are colored grey, while unobserved DTTs are colored white.

bioRxiv preprint doi: https://doi.org/10.1101/2025.05.23.655664; this version posted July 16, 2025. The copyright holder for this preprint (which was not certified by peer review) is the author/funder, who has granted bioRxiv a license to display the preprint in perpetuity. It is made available under aCC-BY 4.0 International license.

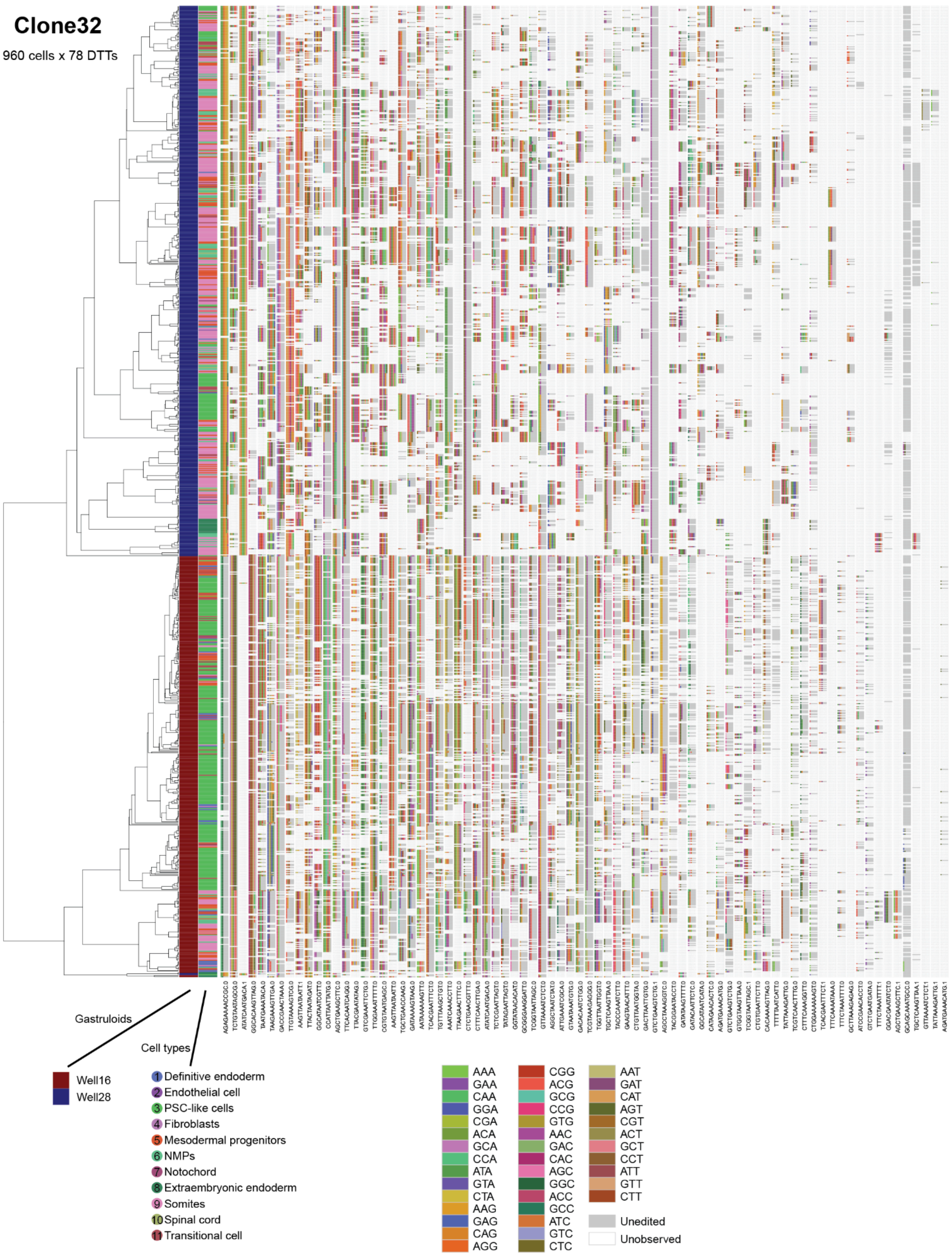

Figure S13. A cell-by-DTT matrix of Clone-32.
Left: The left dendrogram shows the phylogenetic tree of 960 cells from Clone-32 based on cell-cell distances, the colors of the middle bar correspond to two gastruloids to which each cell was assigned, and the colors of the right bar correspond to the 11 annotated cell types. **Right:** Edits at each site across 78 DTTs, ordered by total number of edited sites from high (left) to low (right). Observed, unedited sites are colored grey, while unobserved DTTs are colored white.

bioRxiv preprint doi: https://doi.org/10.1101/2025.05.23.655664; this version posted July 16, 2025. The copyright holder for this preprint (which was not certified by peer review) is the author/funder, who has granted bioRxiv a license to display the preprint in perpetuity. It is made available under aCC-BY 4.0 International license.

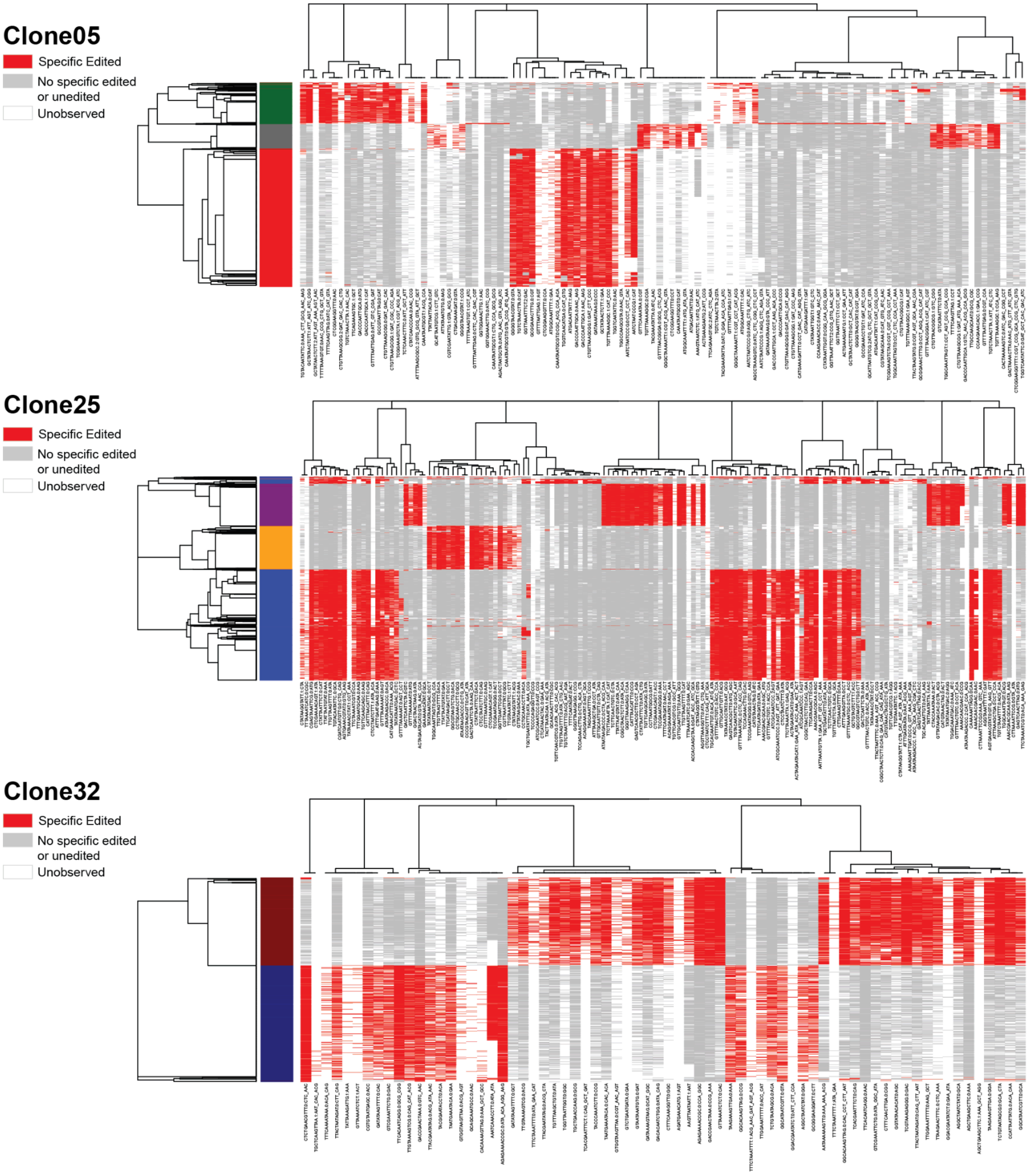

Figure S14. Each monoclonal gastruloid is defined by edits that likely occurred prior to the seeding of its founding mESC.

As shown in **Figure 3A**, we induced PEmax by applying 100 ng/mL doxycycline beginning 24 hrs prior to sorting and seeding of single cells to individual wells. Here we show a biclustered heatmap of 114 (Clone-05), 154 (Clone-25), and 70 (Clone-32) TapeBC-Site-SpecificEdit combinations (columns), after excluding unobserved DTTs, that were detected in >95% of cells assigned to one of the ten major clades shown in **Figure 3D**, and <5% of cells of clades dominated by a different gastruloid from the same clone (rows). We detect 20 to 57 such TapeBC-Site-SpecificEdit combinations per major clade, which we infer to be editing events that occurred during the 24 hr window during which PEmax was induced but prior to seeding of single cells to individual wells.

bioRxiv preprint doi: https://doi.org/10.1101/2025.05.23.655664; this version posted July 16, 2025. The copyright holder for this preprint (which was not certified by peer review) is the author/funder, who has granted bioRxiv a license to display the preprint in perpetuity. It is made available under aCC-BY 4.0 International license.

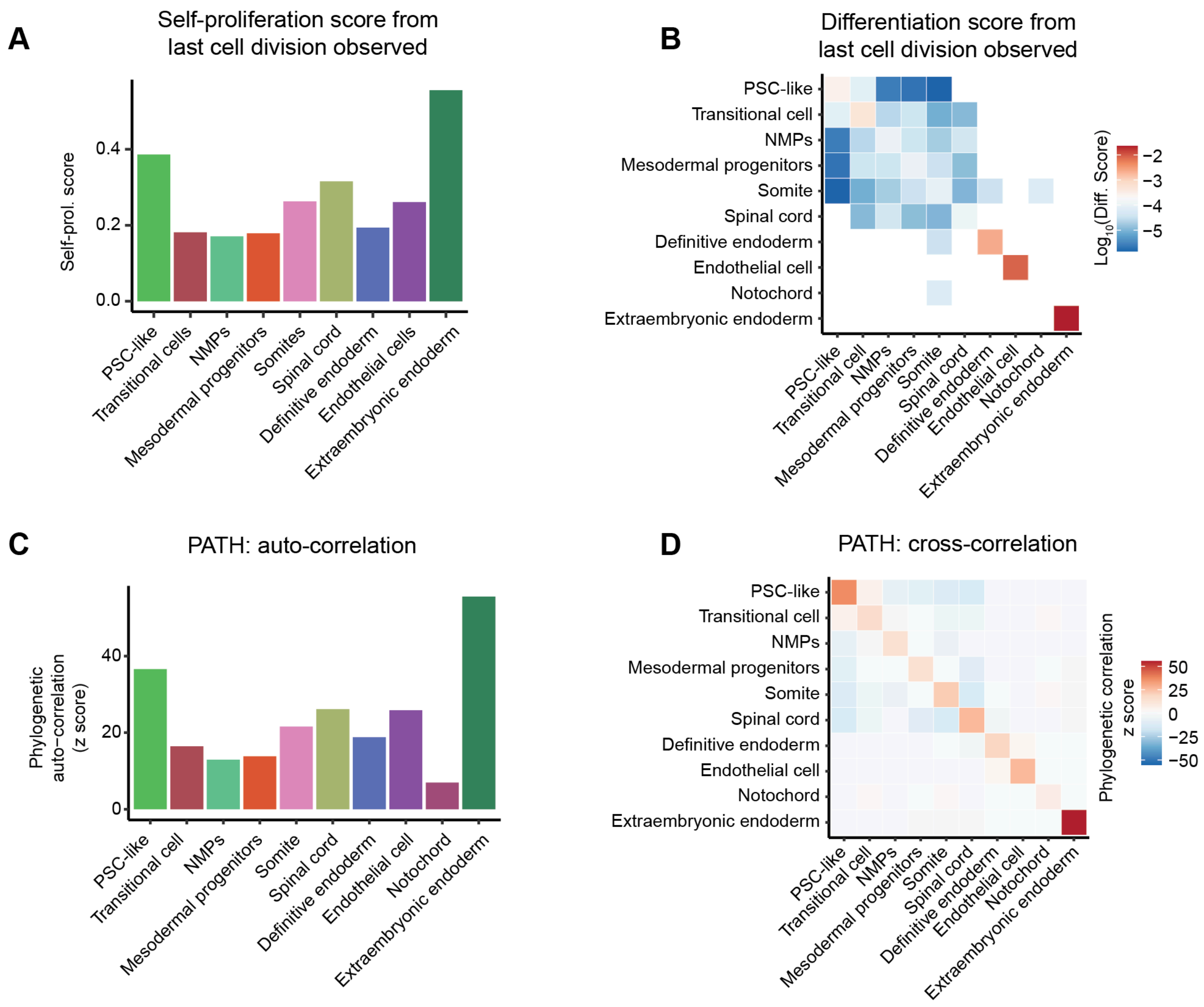

Figure S15. Quantitative assessment of cell-type self-proliferation, differentiation, and correlation in developmental lineages.

The cell-cell distance matrices for the three clones were first combined by manually assigning the maximum distance between cells from different clones. **(A)** The self-proliferation score for each cell type was calculated as the ratio of the frequency of both cells from the last observed cell division event belonging to that cell type to the total number of cells of that type. **(B)** The differentiation score for each possible cell-type-pair was calculated as the ratio of the frequency of the pair detected from the last observed cell division event to the total frequency of all possible cell-cell combinations in the phylogenetic tree. **(C)** The auto-correlation z-score of each cell-type was calculated using PATH[37]. **(D)** The cross-correlation z-score of each possible cell-type-pair was calculated using PATH[37].

bioRxiv preprint doi: https://doi.org/10.1101/2025.05.23.655664; this version posted July 16, 2025. The copyright holder for this preprint (which was not certified by peer review) is the author/funder, who has granted bioRxiv a license to display the preprint in perpetuity. It is made available under aCC-BY 4.0 International license.

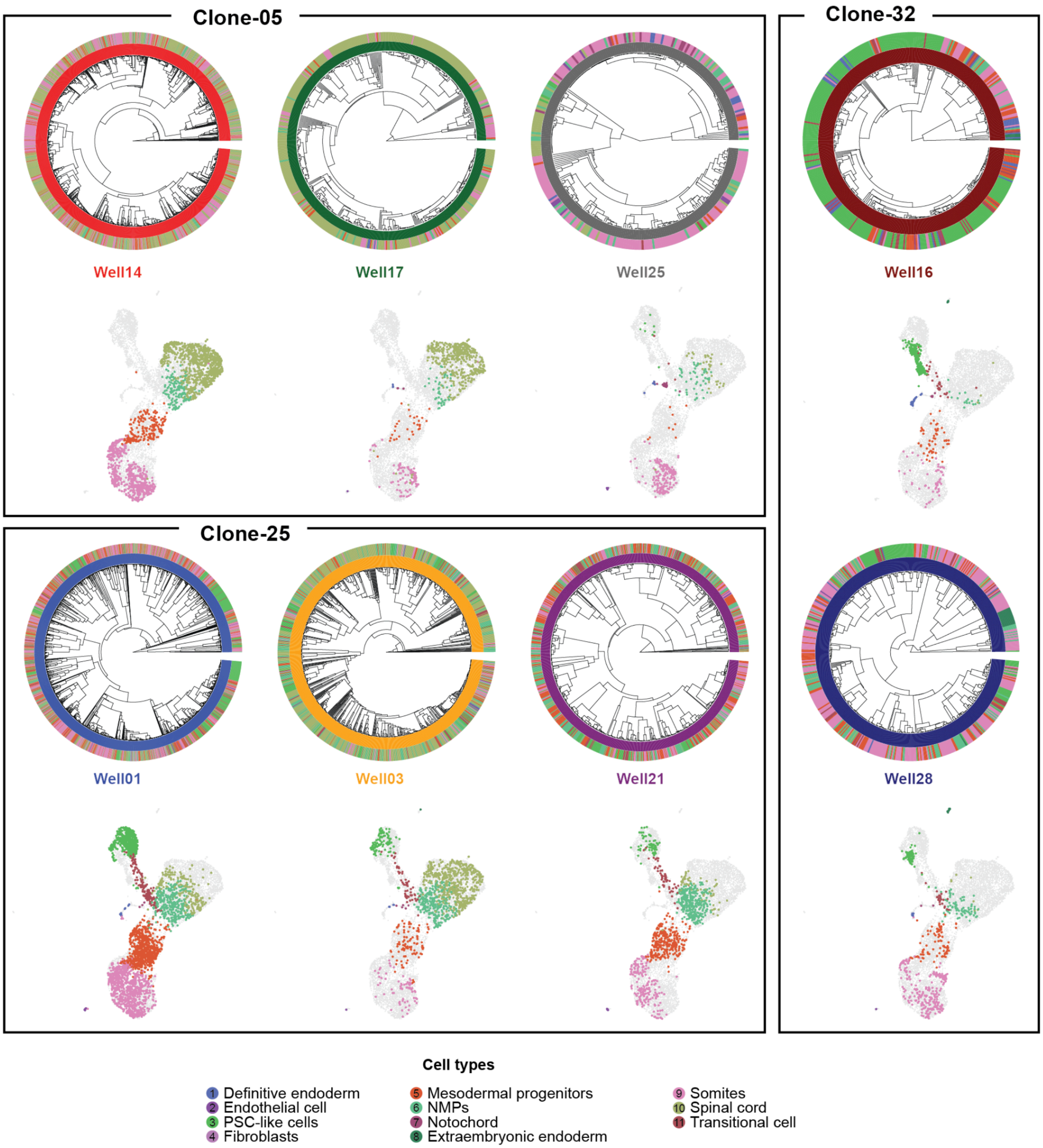

Figure S16. Cell lineage reconstructions of individual monoclonal gastruloids.
For each monoclonal gastruloid: Top: Phylogenetic reconstruction of cell lineage relationships among higher-quality, gastruloid-assigned cells for each gastruloid. The inner circle shows the phylogenetic tree based on cell-cell edit distances, the colors of the middle circle correspond to 8 gastruloids to which each cell was assigned, and the colors of the outer circle correspond to the 11 annotated cell types. Bottom: 2D UMAP, reproduced from **Figure 3B**, showing cells assigned to individual gastruloids, colored by cell types. Gastruloids are grouped by their originating clonal cell line (*i.e.* Clone-05, Clone-25, Clone-32).

bioRxiv preprint doi: https://doi.org/10.1101/2025.05.23.655664; this version posted July 16, 2025. The copyright holder for this preprint (which was not certified by peer review) is the author/funder, who has granted bioRxiv a license to display the preprint in perpetuity. It is made available under aCC-BY 4.0 International license.

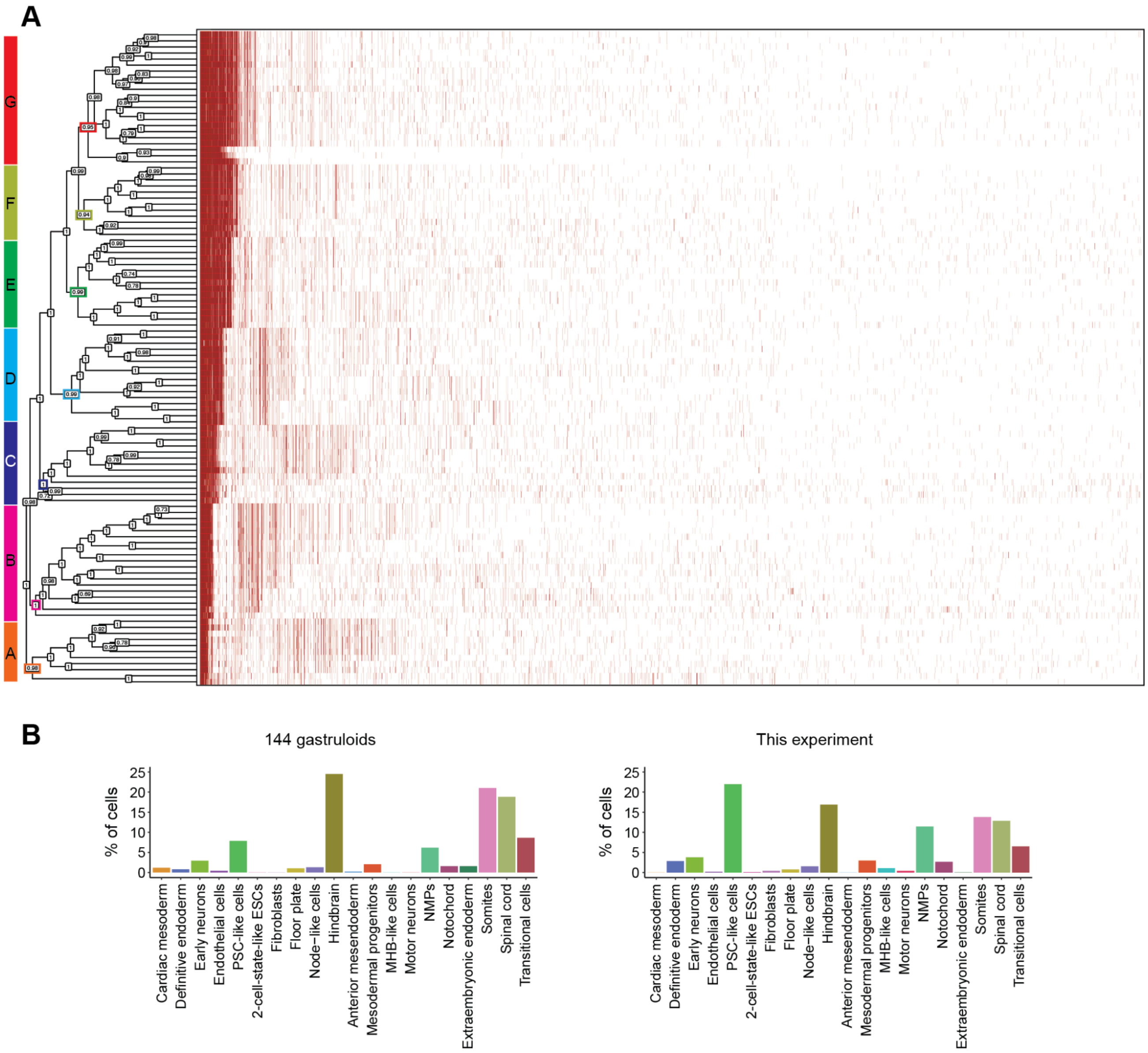

Figure S17. Phylogenetic tree of 108 gastruloids constructed using Debris-seq data.
(A) For each gastruloid, the percentage of reads corresponding to specific edits at six sites across 76 DTTs was determined. Combinations with at least 20% of reads were identified as dominant "TapeBC-Site-Edit" combinations. The Jaccard similarity between the sets of dominant combinations was calculated, and (1 - similarity) was used as the distance metric across gastruloids. The left phylogenetic tree displays the relationships among the 108 gastruloids, based on 7,146 "TapeBC-Site-Edit" combinations shown on the right (red indicates detection in the corresponding gastruloid). The tree also includes the TBE for each ancestral node, calculated from 100 bootstraps. 106 of the 107 ancestor nodes (99%) showed moderate to strong support, with a TBE greater than 70%. The seven major clades (A-G) are highlighted on the left. **(B)** Comparison of the cell type compositions from our earlier experiment involving 144 gastruloids (247,064 cells; **Figure 2B**) and this experiment with 108 gastruloids (66,978 cells; **Figure 5C**).

bioRxiv preprint doi: https://doi.org/10.1101/2025.05.23.655664; this version posted July 16, 2025. The copyright holder for this preprint (which was not certified by peer review) is the author/funder, who has granted bioRxiv a license to display the preprint in perpetuity. It is made available under aCC-BY 4.0 International license.

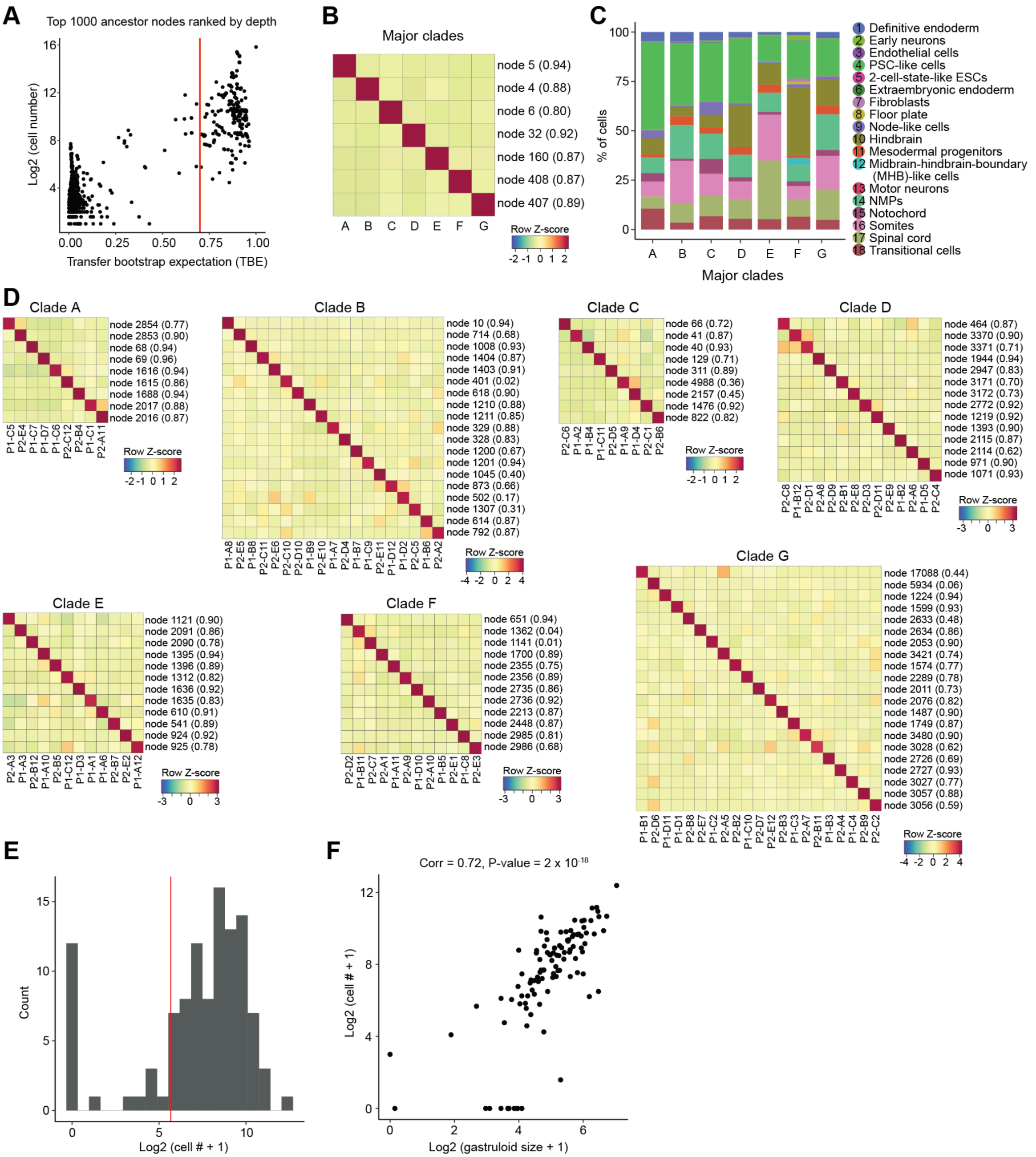

Figure S18. Identifying gastruloid founders among pseudo-ancestors.

(A) We performed a bootstrapping analysis to evaluate the robustness of the top 1,000 clades (ranked by their distance to the root) in the tree shown in **Figure 5D**. For each of 100 bootstraps, an equal number of DTTs (each containing six edit sites) was resampled with replacement to reconstruct a new tree. Clade concordance was determined by comparing each

bioRxiv preprint doi: https://doi.org/10.1101/2025.05.23.655664; this version posted July 16, 2025. The copyright holder for this preprint (which was not certified by peer review) is the author/funder, who has granted bioRxiv a license to display the preprint in perpetuity. It is made available under aCC-BY 4.0 International license.

bootstrap tree to the original and calculating transfer bootstrap expectation (TBE)[35]. Here we show a scatter plot of TBE against the number of cells for each clade. Dashed red vertical line indicates a TBE cutoff of 0.7, with 45% of clades having a TBE > 0.7. **(B)** To assign each of the seven major clades from the "tree of trees" (**Figure 5B**) to a pseudo-ancestor in the single-cell phylogenetic tree (**Figure 5D**), we constructed a cell × "TapeBC-Site-Edit" combination matrix (where 1 indicates the combination was detected in the cell and 0 was not) and normalized it by the sum of each column. For each major clade in the "tree of trees", we identified its exclusively dominant "TapeBC-Site-Edit" combinations. For each pseudo-ancestor in the single-cell phylogenetic tree, we selected the corresponding subset of cells and calculated the fold-change between the mean value of dominant combinations for a given major clade and the mean value of dominant combinations across the other six major clades. For each major clade, we selected all pseudo-ancestors with a ratio of log2(fold-change + 1) between the given clade and the top one among the left six clades >2, and the one with the highest number of cells was designated as the representative of the major clade. The heatmap represents the log2(fold-change + 1) for each of the seven major clades and their assigned pseudo-ancestors, with the TBE for each node based on 100 bootstraps indicated in the brackets. **(C)** After assignment, cell type composition for each major clade. **(D)** For each of the seven major clades, we subset the cells from its assigned pseudo-ancestor and the gastruloids within that clade, then repeated the assignment. For each gastruloid, we identified its exclusively dominant "TapeBC-Site-Edit" combinations. Then, for each pseudo-ancestor, we calculated the fold-change between the mean value of dominant combinations for a given gastruloid and the mean across other gastruloids. For each gastruloid, we selected all pseudo-ancestors with a ratio of log2(fold-change + 1) between the given gastruloid and the top one among the left gastruloids >2, and the one with the highest number of cells was designated as the representative of the gastruloids. During this process, a total of 96 gastruloids were assigned distinct pseudo-ancestors, comprising 51,963 cells. One gastruloid was excluded due to the absence of exclusive dominant combinations, and 11 were excluded because they could not be assigned to a distinct pseudo-ancestor. The heatmap represents the log2 (fold-change + 1) for each gastruloid and their assigned pseudo-ancestors, with the TBE for each node based on 100 bootstraps indicated in the brackets. **(E)** A total of 51,963 cells were assigned to 108 wells/gastruloids, with 88 of the 108 wells/gastruloids having at least 50 assigned cells (indicated by the red vertical line). **(F)** The correlation between the number of cells assigned to individual wells/gastruloids (y-axis) and gastruloid size (x-axis) across all 108 wells/gastruloids. Pearson correlation coefficients and p-values are indicated above the plot. Sizes of individual gastruloids were measured in units of pixels^2 from their brightfield images. A threshold was applied to each image to distinguish the gastruloid from the background.

bioRxiv preprint doi: https://doi.org/10.1101/2025.05.23.655664; this version posted July 16, 2025. The copyright holder for this preprint (which was not certified by peer review) is the author/funder, who has granted bioRxiv a license to display the preprint in perpetuity. It is made available under aCC-BY 4.0 International license.

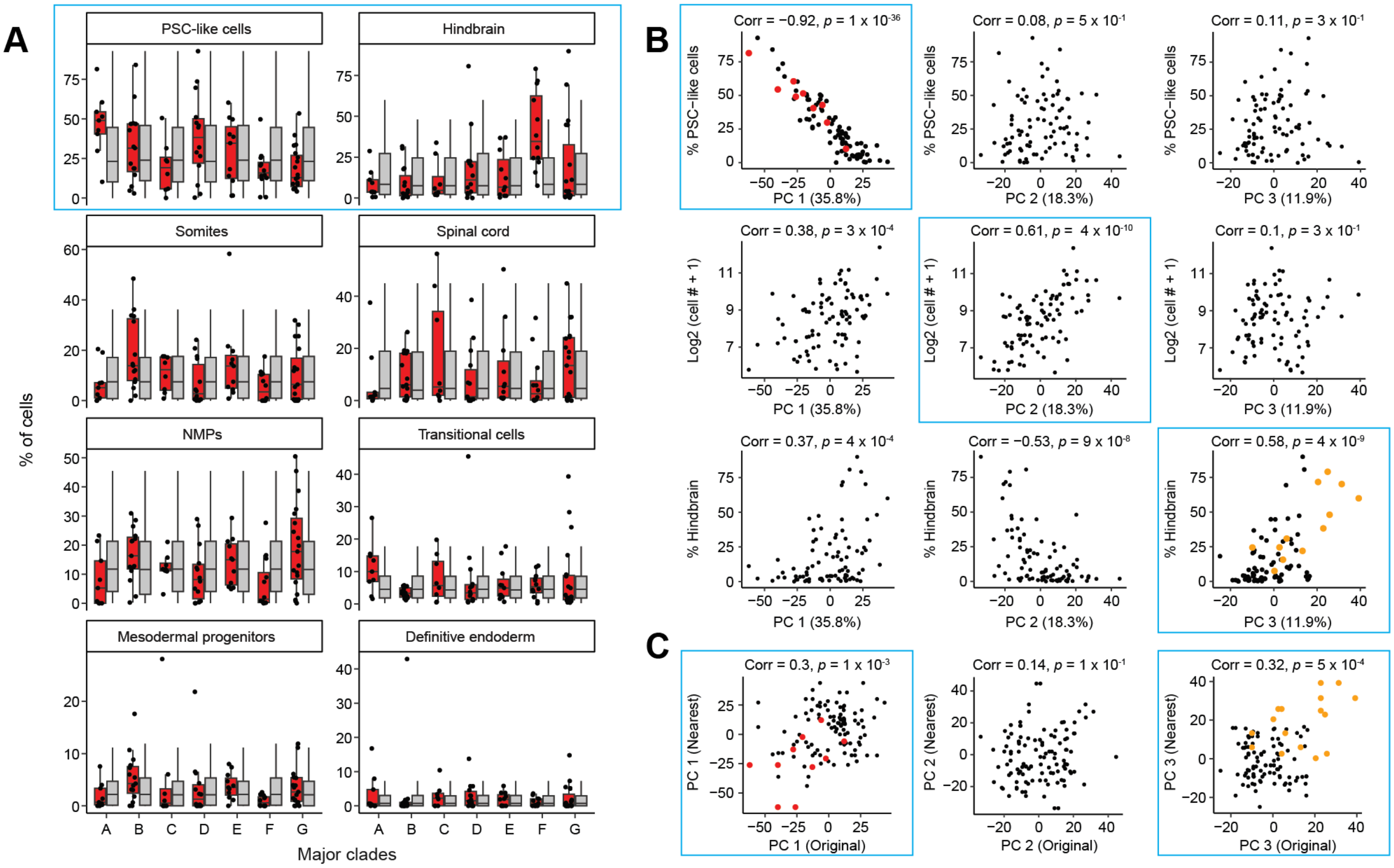

Figure S19. Cell type proportions and PCA analysis of monoclonal gastruloids.
(A) The proportions of the eight most frequent cell types for 88 individual gastruloids (≥50 cells assigned) were calculated, categorized into seven groups corresponding to the major clades, as shown in **Figure 5B**. The group labels for each gastruloid were permuted 5,000 times, and cell-type proportions were recalculated each time. Boxplots, showing 88 replicates from real observations (red box) and 5,000 × 88 replicates from permutation (grey box) in each subpanel, represent the interquartile range (25th, 50th, 75th percentiles), with whiskers extending to 1.5× the IQR. Black dots represent replicates from real observations. **(B)** Embeddings of pseudo-bulk RNA-seq profiles of 88 monoclonal gastruloids (≥50 cells assigned) were generated by aggregating single-nucleus data and performing PCA for dimensionality reduction. The percentage of cells corresponding to selected cell types (1st and 3rd rows), or the cell number (2nd row) is plotted along PC1 (left column), PC2 (middle column), and PC3 (right column). In the top left and bottom right subpanels, gastruloids from major clade A (red) and clade F (orange) were highlighted. **(C)** For each of the 88 gastruloids, their nearest neighbors were identified from the phylogenetic lineage tree, resulting in 112 pairs. Of note, some gastruloids had more than one nearest neighbor, and some pairs were redundant (both A vs. B and B vs. A were retained). For each of the top three PCs, the values for each gastruloid (x-axis) are plotted against those of their nearest neighbors (y-axis). Pearson correlation coefficients and p-values are indicated above each plot. Gastruloids from major clade A (red) and clade F (orange) are colored. Of note, subpanels highlighted with a light blue rectangle are shown in **Figure 5E-G** as well.

bioRxiv preprint doi: https://doi.org/10.1101/2025.05.23.655664; this version posted July 16, 2025. The copyright holder for this preprint (which was not certified by peer review) is the author/funder, who has granted bioRxiv a license to display the preprint in perpetuity. It is made available under aCC-BY 4.0 International license.

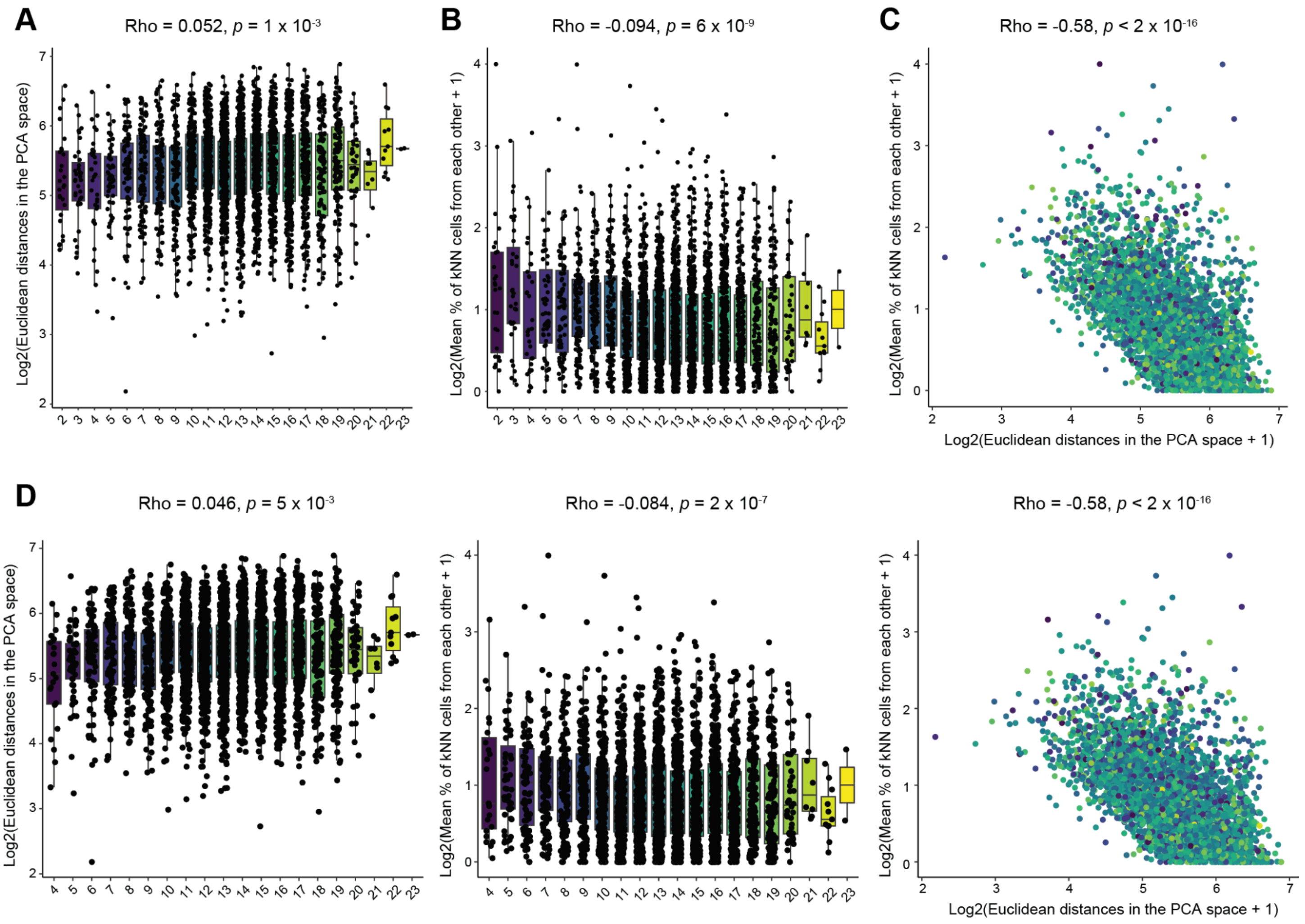

Figure S20. Pairwise distance and neighboring cell relationships among monoclonal gastruloids.
(A) Pairwise Euclidean distances among 88 monoclonal gastruloids (≥50 cells assigned), calculated in 10-dimensional PCA space, are plotted against the "first epoch" lineage distances of their founder mESCs. Boxplots, encompassing a total of 3,828 pairs, represent IQR (25th, 50th, 75th percentile) with whiskers representing 1.5× IQR. Spearman correlation coefficient and p-value are indicated above. **(B)** For each gastruloid, the top 15 nearest neighboring cells for its cells were identified based on the 30-dimensional PCA space of the single-cell transcriptome. These neighboring cells were categorized by their gastruloid of origin, normalized by the total number of neighboring cells from that gastruloid, across all gastruloids. For each pair of gastruloids (e.g., A and B), the percentages of neighboring cells of A originating from B and those of B originating from A were averaged. Pairwise percentages of neighboring cells from each other among 88 gastruloids, are plotted against their lineage distances. Boxplots, encompassing a total of 3,828 pairs, represent IQR (25th, 50th, 75th percentile) with whiskers representing 1.5× IQR. Spearman correlation coefficient and p-value are indicated above. **(C)** The log2 pairwise Euclidean distances are plotted against the log2 pairwise percentages of neighboring cells for all 3,828 pairs. Spearman correlation coefficient and p-value are indicated above. **(D)** For each of the 88 gastruloids, the nearest neighbors were identified from the phylogenetic lineage tree, yielding 112 pairs. After removing redundancies (e.g., retaining only one of A vs. B and B vs. A), 76 pairs remained. These 76 pairs were excluded, and the analyses shown and described in panels **A–C** were repeated with the remaining 3,752 pairs.
